# Supplementary material for: Immune Modulation Ability of Hepcidin from Teleost Fish
Source: Animals (Basel). 2022 Jun 20;12(12):1586. doi: 10.3390/ani12121586 (PMC9219549; doi:10.3390/ani12121586)
Supplement: Supplementary file 1 [file animals-12-01586-s001.zip › animals-1759094-supplementary.pdf]

Table S1. Primer sequences used in this study

**Species**	Molecule	**Primer**	**Sequence**
D. labrax	Interleukin 1β	Forward	5'-ATTACCCACCACCCACTGAC-3'
		Reverse	5'-TCTCTTCCACTATGCTCTCCAG-3'
	Interleukin 10	Forward	5'-ACCCCGTTCGCTTGCCA-3'
		Reverse	5'-CATCTGGTGACATCACTC-3'
	tumor necrosis factor α	Forward	5'-GCCAAGCAAACAGCAGGAC-3'
		Reverse	5'-ACAGCGGATATGGACGGTG-3'
	β-Actin	Forward	5'-ATGTGGATCAGCAAGCAGG-3'
		Reverse	5'-AGAAATGTGTGGTGTGGTCG-3'
O. mykiss	Interleukin 1β	Forward	5'-GTCACATTGCCAACCTCATCATCG-3'
		Reverse	5'-GTTGAGCAGGTCCTTGTCCTTGAA-3'
	Interleukin 10	Forward	5'-GCTATGGACAGCATCCTGAAGT-3'
		Reverse	5'-CGATGGAGTCGATGGGAGATTTA-3'
	tumor necrosis factor α	Forward	5'-CTGTGTGGCGTTCTCTTAATAGCAGCTT-3'
		Reverse	5'-CATTCCGTCCTGCATCGTTGC-3'
	ELF-1α	Forward	5'-GTCTACAAAATCGGCGGTAT-3'
		Reverse	5'-CTTGACGGACACGTTCTTGA-3'
